# Th17/Treg imbalance in patients with severe acute pancreatitis: Attenuated by high-volume hemofiltration treatment

**DOI:** 10.1097/MD.0000000000021491

**Published:** 2020-07-31

**Authors:** Jiguang Guo, Zhen Li, Dan Tang, Jianbin Zhang

**Affiliations:** a Department of Nephrology,People's Hospital of Rongchang District; b Department of Nephrology, Yongchuan Hospital of Chongqing Medical University; c Department of Nephrology, Yongchuan Hospital of traditional Chinese Medicine, Chongqing, China.

**Keywords:** high-volume hemofiltration, multiple organ dysfunction syndrome, severe acute pancreatitis, Th17 cells, Treg cells

## Abstract

**Background:**

To investigate the effect of high-volume hemofiltration (HVHF) on Th17/Treg imbalance in patients with severe acute pancreatitis (SAP).

**Methods:**

Forty-two patients with SAP were randomly received 24 hours of continuous HVHF (n = 21) or without HVHF (n = 21). At day 28, all 42 patients were divided into survival group (n = 32) and non-survival group (n = 10). Venous blood samples collected at 0, 6, 12, and 24 hours during HVHF treatment (or equivalent time in non-HVHF group) were assessed by flow cytometry to detect Th17 and Treg cells. Concentrations of IL-6, IL-17, IL-10, and TGF-β1 were detected by enzyme-linked immunosorbent assay.

**Results:**

Th17%, Treg%, Th17/Treg, and levels of related cytokines were significantly higher in SAP patients than healthy controls (*P* < .05), and these changes were more pronounced in SAP patients with multiple organ failure than those with single organ failure (*P* < .05). After HVHF treatment, Th17%, Treg%, Th17/Treg, IL-6, IL-17, and IL-10 significantly reduced (*P* < .05), while there were no significant changes in non-HVHF group (*P* > .05). In addition, acute physiology and chronic health evaluation II and sequential organ failure assessment scores decreased markedly after HVHF treatment. Baselines of Th17%, Treg%, Th17/Treg, and related cytokines were significantly higher in non-survival group than survival group. Both acute physiology and chronic health evaluation I score and IL-6 level were positively correlated with Th17% before and after HVHF treatment (*P* < .01).

**Conclusions:**

Th17/Treg imbalance is present in SAP and may be correlated with its severity and prognosis. HVHF effectively attenuates the Th17/Treg imbalance in SAP patients. The beneficial effect of HVHF on Th17/Treg imbalance is possibly associated with removing excess inflammatory mediators.

## 1 Introduction

Although progress continues to be made in the treatment and management of severe acute pancreatitis (SAP), the mortality of SAP associated with multiple organ dysfunction syndrome (MODS) can reach up to 36% to 50%.^[1]^ It is widely accepted that local pancreatic inflammation caused by many factors is the initiator of systemic inflammatory response syndrome (SIRS). This in turn results in the development of overwhelming systemic inflammation and organ failure, ultimately contributing to deaths. So, recognition of the immune dynamic changes in the progress of SAP is essential for early goal-directed therapy.

Th17 cells, first described in mice in 2005 as the cellular source of IL-17, participating in inflammatory reactions and autoimmune diseases, represent another novel CD4+ T cell subset different from Th1 and Th2 cells.^[2]^ Treg cells, initially called “suppressor cells,” play an important role in anti-inflammation reactions and autoimmune diseases by releasing anti-inflammatory cytokines such as IL-10 and TGF-β1.^[3,4]^ Similar to Th1 and Th2, Th17 and Tregs interdependently regulate the differentiation of one another to maintain immunological balance.^[5]^ The balance between Th17 and Treg cells is significant in the prevention of inflammatory and autoimmune diseases.^[6]^ However, little is known about Th17 and Treg cells in SAP patients. Thus, we presume that Treg/Th17 imbalance may exist in SAP patients and correlate closely with progress of SIRS and MODS.

Continuous blood purification showed significant improvement in the treatment of acute renal failure, acute respiratory distress syndrome, sepsis, and MODS.^[7]^ Early blood purification can ameliorate immune function and maintain homeostasis by removing inflammatory mediators such as TNF, IL-10, IL-6, and IL-8 in patients with sepsis.^[8]^ Considering the cascading effect of inflammation in SAP, high-volume hemofiltration (HVHF) treatment in SAP patients has increased progressively. Early preemptive application of hemofiltration is feasible and essential in the treatment of SAP.^[9]^ Our study was conducted to test changes of Th17%, Treg%, and related cytokines during HVHF in SAP patients and evaluate the efficacy of hemofiltration.

## 2 Materials and methods

### 2.1 Patients and groups

Forty-two SAP patients (including 26 males and 16 females ranged from 24 to 67 years old) admitted to the intensive care unit of our hospital from September 2015 to February 2017 were selected as study group if they had displayed symptoms for no more than 48 hours, together with twenty healthy volunteers as control group. The diagnosis of SAP was based on Atlanta criteria.^[10]^ Twenty-one patients were randomly selected for HVHF and conventional treatment (HVHF group) while the remaining patients received only conventional treatment (non-HVHF group) including ventilation, adequate hydration, and inotropic support. Treatment was started within 4 hours after fulfillment of SAP criteria. All patients were divided into 2 groups according to severity: group A (with single organ failure, n = 14) and group B (with multiple organ failure, n = 28). Multiple organ failure was diagnosed if dysfunction of more than 1 organ was detected, and SOFA (sepsis-related organ failure assessment) score was calculated for assessment of organ dysfunction.^[11,12]^ The total cohort was also divided into survival group (n = 32) and non-survival group (n = 10) for analysis. Exclusion criteria:

(1) age <18 years or >80 years;
(2) autoimmune diseases (such as rheumatoid arthritis, multiple sclerosis, systemic lupus erythematosus, or asthma);
(3) malignant tumors, acquired immune deficiency syndrome, and other underlying diseases;
(4) use of hormones or immunosuppressors within the past 3 months before hospitalization; and
(5) unexpected termination of HVHF treatment.

The demographic and clinical characteristics of study population are shown in Tables 1 and 2. The study was granted by our hospital and ethics approval was obtained from the medical ethics committee. Signed informed consent documents were obtained from healthy volunteers, patients, or their legally authorized representatives.

**Table 1:**
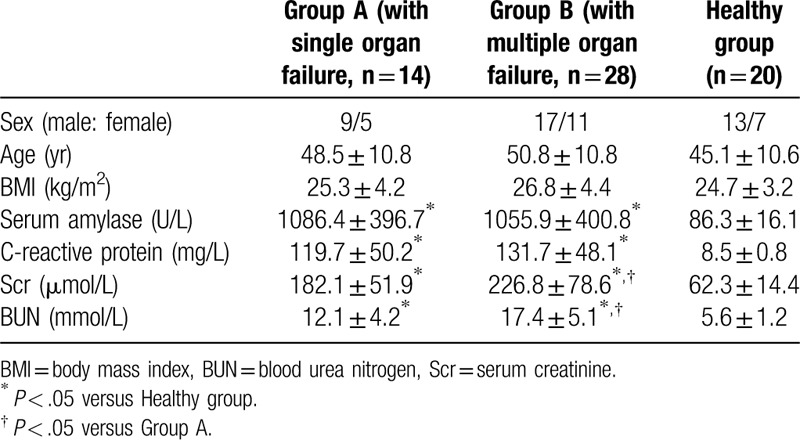
Baseline characteristics of the study population.

**Table 2:**
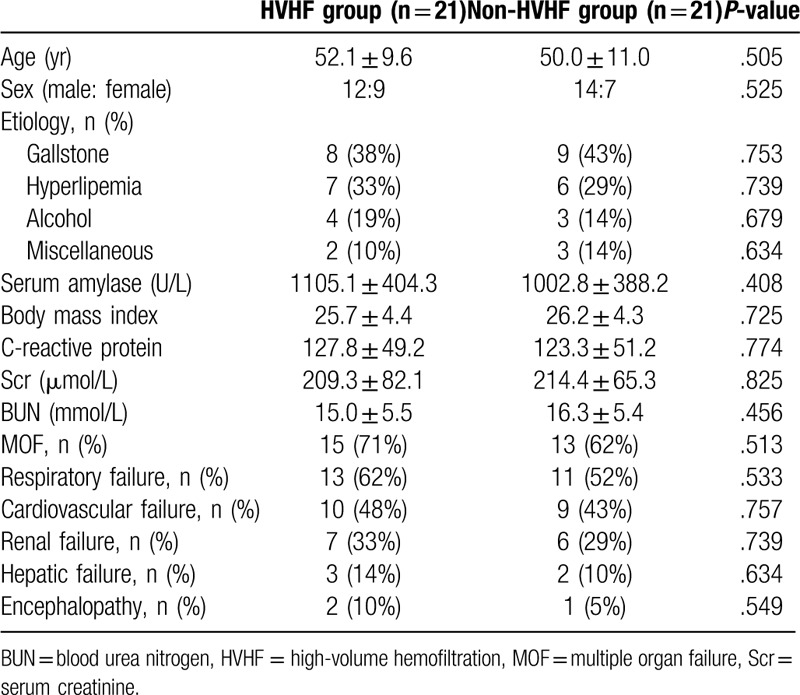
Baseline characteristics of the HVHF group and non-HVHF group.

### 2.2 HVHF settings

A 14F double lumen catheter was catheterized in the internal jugular or femoral vein to establish vascular access. Continuous HVHF was conducted using the Aquarius hemofiltration machine (Baxter Health, Deerfield, IL) with a high-flux polysulfone membrane blood filter (AV600S, 1.6 m^2^, 35 kD limit; Fresenius Medical Care, Bad Homburg, Germany). Blood flow rate was kept between 250 and 300 mL/min and substitution fluid was infused at 70 mL/kg/h with a pre-dilution method. The hemofilter was replaced every 24 hours and the whole process of HVHF therapy lasted for 48 hours. The filter performance was assessed by monitoring fluid urea nitrogen/blood urea nitrogen ratio at initiation and every 12 hours. Low molecular heparin was used for anticoagulation at an initial dose of 60 U/kg and a maintenance dose of 6 U/kg/h. Activated clotting time in the systemic circulation was maintained between 150 and 180 seconds and checked every 6 hours. Patients who developed coagulation disorders or active bleeding were applied to no-heparin hemofiltration and the hemofilter was cleaned by 0.9% sodium chloride every 30 minutes. We monitored the central venous pressure to manage fluid balance and adjust Quf in patients who were fluid overloaded.

### 2.3 Specimen collection and preparation

In HVHF group, patients were connected to the blood filtration machine within 6 hours after admission, and venous blood samples (5 mL) were collected at 0 hours (before HVHF), 6, 12, and 24 hours. Samples (5 mL) from non-HVHF patients were collected at equivalent time points during routine treatment within 6 hours of admission. During the same period, isovolumetric blood samples were drawn from healthy volunteers in the physical examination center of our hospital. All samples were refrigerated at 4°C after EDTA anticoagulation. Serum and peripheral blood mononuclear cells were separated by density gradient centrifugation at 2000 rpm for 20 minutes. The former was stored at −80°C for subsequent cytokine detection and the latter was adjusted to 1 × 10^6–8^/mL in RPMI 1640 medium supplemented with 100 U/mL penicillin, 100 μg/mL streptomycin, 2 mM/L glutamine, and 10% fetal calf serum (Gibco BRL). The cell suspension was then seeded into 24-well cell culture plates. Cells were treated with phorbol myristate acetate (Abcam; 50 ng/mL), ionomycin (Abcam; 2 g/mL), and brefeldin A (Selleck Chemicals, Houston, TX; 3 g/mL), and incubated in the dark at 37°C under 5% CO_2_ atmosphere for 5 hours.

### 2.4 Flow cytometry for Th17 cell detection

The stimulated cells were harvested, centrifuged, resuspended in PBS, and then incubated with 5 uL anti-human CD4-FITC (eBioscience) at room temperature in the dark for 20 minutes. After washing with fresh PBS twice, cells were incubated with 100 uL FIX & PERM medium A (Invitrogen) for 15 minutes. Following centrifugation and resuspension, cells were treated with 100 uL FIX & PERM medium B (Invitrogen) and incubated in the dark for 20 minutes. Then 5 uL anti-human IL-17-PE was added (eBioscience) to the suspension. A parallel control group was treated instead with 5 uL anti-mouse PE-anti-IgG1. Both suspensions were incubated at room temperature in the dark for 15 minutes. Cells were then washed, centrifuged, suspended in PBS, and then analyzed by flow cytometry.

### 2.5 Flow cytometry for Treg cell detection

The stimulated cells were treated with 5 uL anti-human CD4-FITC and 5 L anti-human CD25-PE (eBioscience), and then incubated in the dark for 30 minutes. After being treated with 500 uL FOXP3 fixation/permeabilization working solution (eBioscience) and 1 mL permeabilization buffer (eBioscience) sequentially, the suspension was incubated with 5 uL anti-human FOXP3-APC. In the parallel control group, 5 uL anti-mouse APC-anti-IgG1 (eBioscience) was added. Both solutions were incubated at room temperature in the dark for 30 minutes. Finally, cells were washed, centrifuged, suspended in PBS, and then subjected to flow cytometry.

### 2.6 Detection of plasma cytokines

Plasma levels of IL-17, IL-6, IL-10, and TGF-β1 were measured by enzyme-linked immunosorbent assay kits (R&D) according to the manufacturer's instructions. The minimum detectable concentrations were 15 pg/mL (IL-17), 0.7 pg/mL (IL-6), 3.9 pg/mL (IL-10), and 15.4 pg/mL (TGF-β1), respectively. All measurements were performed in duplicate.

### 2.7 Prognostic indices

Before and after 24 and 48 hours of treatment, APACHE II and SOFA scores were used to assess the patient conditions. These evaluations were performed in intensive care unit by 1 physician who was not blinded to treatment modes. The primary endpoint was mortality on day 28.

### 2.8 Statistical analyses

Measurement data are expressed as mean ± standard deviation. Two group means were compared by *t* test and multiple group means were compared by ANOVA. Enumeration data were compared by using *χ*^2^ test or Fisher exact test. We used Kaplan–Meier curve to compare survival in the 2 groups for the first 28 days after treatment. Pearson coefficient was used to measure correlation between variables. Two-tailed *P* < .05 was considered statistically significant and all statistical analyses were performed with. SPSS 21.0

## 3 Results

### 3.1 Basic clinical and laboratory characteristics among groups

There were no significant differences in age, sex ratio, body mass index, etiology, CRP, blood urea nitrogen, serum amylase, and number of failure organs on admission between HVHF group and non-HVHF group (Table 1). Between group A and group B, there were no significant differences in age, body mass index, and sex ratio (Table 2).

### 3.2 T lymphocyte frequencies and cytokine concentrations among groups

**Figure 1:**
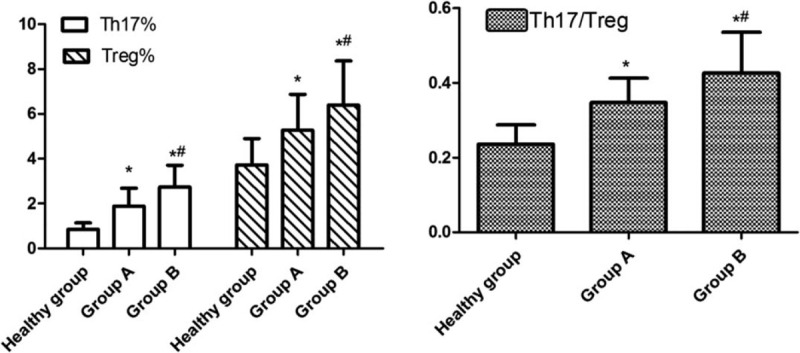
Peripheral blood Th17%, Treg%, and Th17/Treg among groups. ^∗^*P* < .05 versus healthy group, ^#^*P* < .05 versus group A.

**Figure 2:**
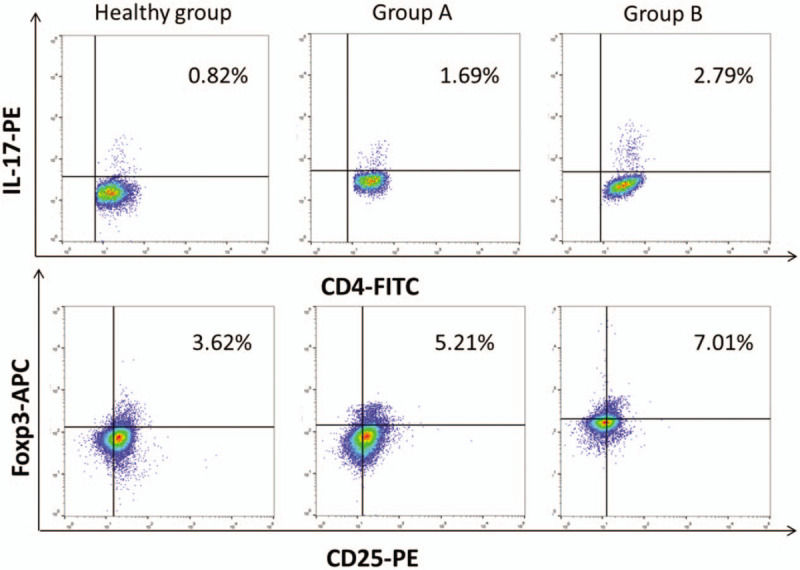
The representative images of flow cytometry analysis of Th17 and Treg cells among groups.

As shown in Table 3 and Figure 1, Th17% (1.89% ± 0.80% and 2.74% ± 0.97% vs 0.85% ± 0.28%), Treg% (5.28% ± 1.60% and 6.14% ± 1.87% vs 3.73% ± 1.17%), and Th17/Treg (0.35 ± 0.065 and 0.45 ± 0.15 vs 0.24 ± 0.052) in group A and group B were higher than those in healthy group. Compared to group A, group B exhibited higher Th17%, Treg%, and Th17/Treg, and the difference in Th17% was more distinct (Fig. 2).

**Table 3:**
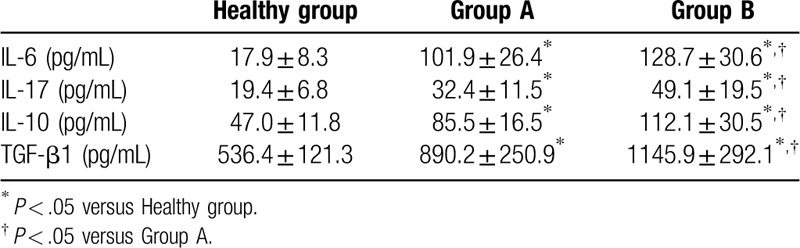
Cytokine concentrations among groups.

### 3.3 Changes in T lymphocyte frequencies and cytokine concentrations during HVHF

Compared to baseline (0 hours), Th17% and IL-10 decreased significantly at 24 hours during HVHF treatment, while Treg%, IL-6, and IL-17 were significant lower at 12 hours and 24 hours of HVHF. In contrast, Th17%, Treg%, and related cytokines did not change significantly over 24 hours in the non-HVHF group (Table 4).

**Table 4:**
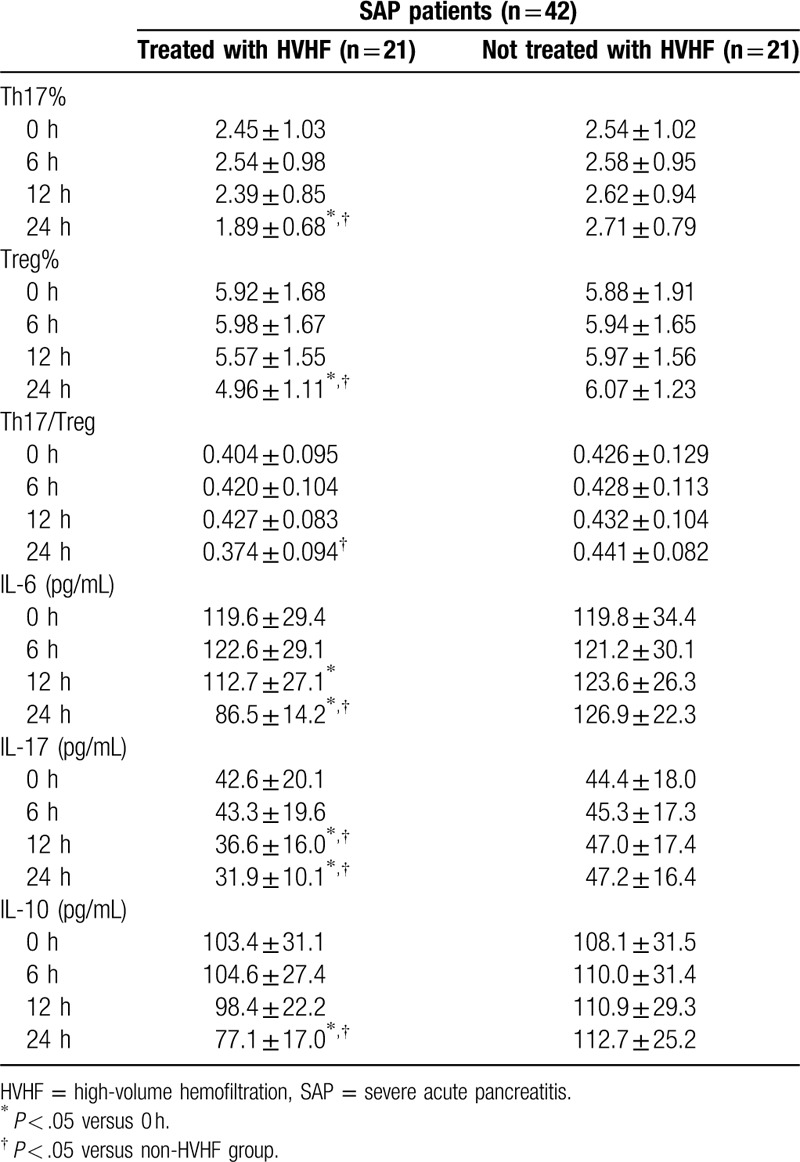
Changes of Th17, Treg, and related cytokine concentrations between HVHF group and non-HVHF group during 24 h treatment.

### 3.4 Changes in APACHE II and SOFA scores

**Figure 3:**
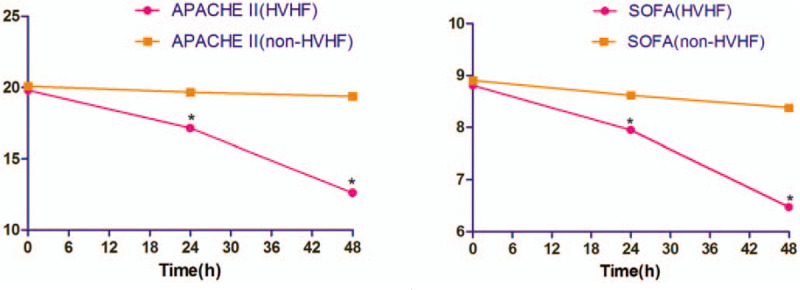
Change of APACHE II and SOFA scores in HVHF and non-HVHF group. ^∗^*P* < .05 versus 0 h. APACHE II = acute physiology and chronic health evaluation II, HVHF = high-volume hemofiltration, SOFA = sequential organ failure assessment.

Both APACHE II and SOFA scores significantly reduced after 24 hours and 48 hours HVHF treatment, while no significant changes occurred in the non-HVHF group (Fig. 3).

### 3.5 Association of leukocyte counts with survival

**Figure 4:**
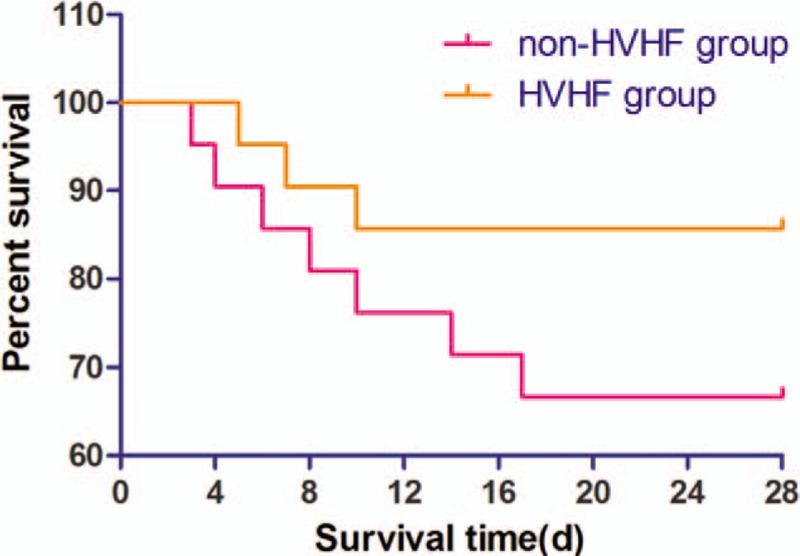
Kaplan–Meier survival curve in HVHF group and non-HVHF group. HVHF = high-volume hemofiltration.

Survival analysis showed that the survival rate on day 28 was 85.7% and 66.7% in the HVHF and non-HVHF group respectively. The difference had statistical significance (*P* < .05) (Fig. 4). Among all SAP patients, Th17%, Treg%, and related cytokine levels were significantly higher in the non-survival group than the survival group (Table 5).

**Table 5:**
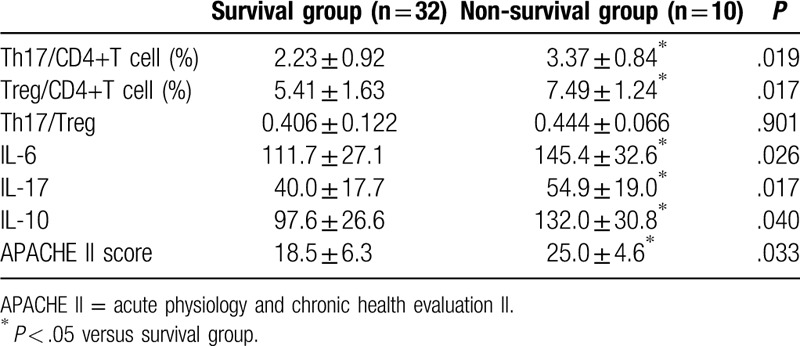
Th17%, Treg%, and related cytokine concentrations between survival and non-survival group.

### 3.6 Correlations between Th17% and APACHEII score and IL-6 during HVHF

**Figure 5:**
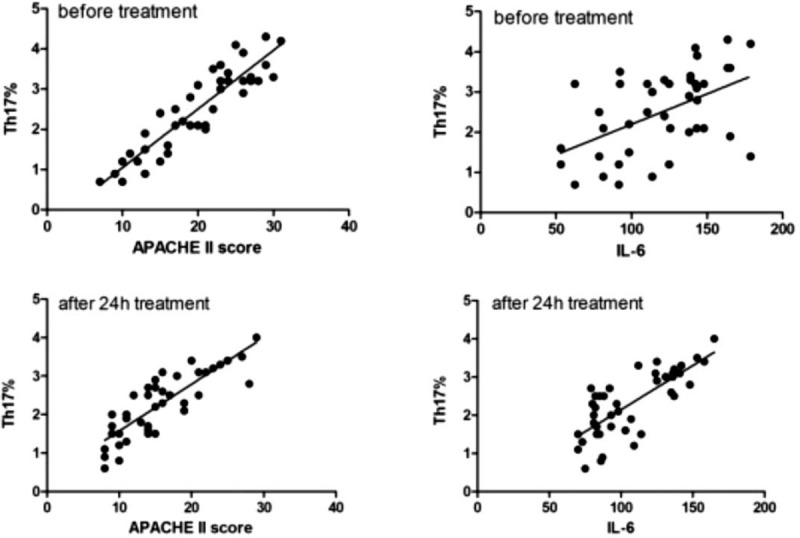
Pearson correlation of Th17% and APACHE II score before and after treatment. APACHE II = acute physiology and chronic health evaluation II.

There was a positive correlation between Th17% and APACHE II score (*r* = 0.912, *P* < .01) and between Th17% and IL-6 concentration (*r* = 0.505, *P* < .01) before treatment. After 24 hours treatment, Th17% was also positively correlated with APACHE II score (*r* = 0.844, *P* < .01) and IL-6 concentration (*r* = 0.759, *P* < .01) (Fig. 5).

## 4 Discussion

SAP characterized by the presence of persistent single or multi-organ failure, is associated with overwhelming systemic inflammation and massive release of inflammatory mediators.^[13]^ Gallstones and alcohol are the most prevalent causes of SAP, and the activation of cascade of digestive enzymes can lead to autodigestive injury. Because of the complexity and differences in SAP process, traditional methods (including Balthazar CT, BISAP, and SOFA scoring system) cannot make early diagnosis and severity grading after admission to hospitals. Some scholars found that lymphocyte ratio is markedly different between SAP and MAP patients and is valuable for the early differential diagnosis of SAP.^[14]^ It is known that Th17 and Treg cells play an essential role in various immune processes, especially inflammation, as well as progression of illness.^[15]^ Loos et al found that Th17 cells play an important role in the pathogenesis of autoimmune pancreatitis.^[16]^ In this study, SAP patients with multi-organ dysfunction exhibited higher Th17% compared with healthy group and single-organ dysfunction group. Further subgroup analysis revealed that Th17% was significantly higher in non-survival group than survival group. These data suggested T lymphocyte-dependent immune dysfunction exists in SAP patients. And we identified Th17% as an effective predictor of disease severity and mortality.

IL-17, mainly secreted by Th17 cells, has been identified to have an important role in the host defense against inflammatory and autoimmune diseases.^[17]^ Some researchers suggested that IL-17 is an predictive marker of early forms of SAP and its concentration is correlated with the risk of organ dysfunction.^[18]^ In our preliminary experiment, we found that high serum IL-17 is a significant risk factor for poor prognoses in SAP patients and associated with bacterial load.^[19]^ Our data showed that related cytokines (IL-6, IL-17, IL-10, and TGF-β1) increased significantly in group B compared to healthy group and group A. In the meantime, patients in non-survival group exhibited higher cytokine concentrations than in survival group. So, we concluded that immune related cytokine concentrations can reflect the severity of SAP. Unlike pro-inflammatory cytokines, IL-10 and TGF-β1 are mainly secreted by Treg cells which inhibit effector T cell and maintain immune tolerance.^[20]^ In the progress of SIRS, the body also accumulates an anti-inflammatory response. This early pro-inflammatory response is then gradually suppressed by the development of the compensatory anti-inflammatory response syndrome.^[21]^ Gunjaca et al confirmed the theory that SIRS and compensatory anti-inflammatory response syndrome are 2 mutually interconnected and simultaneous processes occurred in the acute inflammatory response in patients with acute pancreatitis by means of evaluating pro- and anti-inflammatory cytokines.^[22]^ In our present study, both Treg cell ratio and related cytokines increased significantly in SAP patients, suggesting the accumulation of anti-inflammation effect and the occurrence of immunosuppression. Therefore, we consider that pro-inflammatory effect and anti-inflammatory effect coexist in the development of SAP.

The inflammatory mediators play a crucial role in the cascade amplification and cause the distant organ dysfunction.^[23]^ But the conventional therapy including fluid resuscitation, gastrointestinal decompression, parenteral nutrition, antibiotic, and so on cannot relieve the inflammatory reactions and improve general conditions in a short period, which explains that the mortality associated with multiple organ failure is fairly high. Continuous blood purification was originally used for acute kidney injury, but now it is widely used in critical infection.^[24]^ Wang et al concluded that HVHF therapy is effective in the treatment of hyperlipidemic SAP and can improve prognosis of patients.^[25]^ Some scholars found that pulsed HVHF is a promising technique for SAP patients by maintaining hemodynamics and reducing levels of inflammatory cytokines.^[26]^ In the present study, Th17%, Treg%, and related cytokines decreased significantly after treatment of HVHF, while patients in non-HVHF group showed no such changes over 24 hours, demonstrating that HVHF can remove over-expressed inflammatory cytokines and recover the T lymphocyte balance to some extent in SAP patients. APACHE II score and SOFA score in HVHF group patients decreased significantly after 48 hours treatment, and the survival rate was higher at day 28 in HVHF group, suggesting that HVHF is an effective and feasible treatment for SAP patients by restoring Th17/Treg balance and ameliorating general conditions.

APACHE II score is commonly used to assess the severity of diseases in critically ill patients, and also used as a prognostic indicator.^[27]^ Evidence showed that sepsis patients who survived during hospitalization had lower APACHE II scores compared to non-survival group.^[28]^ IL-6 secreted by many immune cells is a main inflammatory factor in the early stage of infection. Sepsis patients with sustained rising of IL-6 exhibited poor prognosis.^[29]^ Consistently, we found that IL-6 level and APACHE II score were markedly higher in non-survival group than survival group. Moreover, the present data also showed that there is a positive correlation between Th17% and IL-6 concentration and between Th17% and APACHE II score before or after treatment, further supporting the efficacy of Th17 cell ratio as a sensitive early-stage indicator of prognosis in SAP patients. Differentiation of Treg cells is induced by TGF-β and IL-2, while differentiation of Th17 cells is induced by TGF-β and IL-6.^[30,31]^ This process is amplified by the interaction of cytokines secreted by Th17 and Treg cells. Evidence showed that large amounts of cytokines secreted by different kinds of activated lymphocytes were observed in early stage of SAP, however, the absolute number of circulating lymphocyte subsets decreased markedly.^[32,33]^ So, considering the depletion of lymphocytes and accumulation of anti-inflammatory effect, we suppose that SAP patients in the middle-to-late stage are in a state of immunodeficiency or immune paralysis. On the one hand, early HVHF can eliminate the excessive secretion of inflammatory mediators in a short time. On the other, HVHF can reduce the depletion of lymphocyte and improve the immune balance.

This study was conducted in a single center with a small sample size and the efficacy of the continuous HVHF treatment on SAP patients has not been comprehensively assessed. Therefore, future larger sample-sized multicenter studies are expected.

In conclusion, Th17/Treg imbalance is present in SAP and may be correlated with the severity and prognosis of SAP. HVHF effectively attenuates the Th17/Treg imbalance in SAP patients. The beneficial effect of HVHF on Th17/Treg imbalance is possibly associated with removing excess inflammatory mediators. Therefore, it is worthy of clinical promotion and application.

## Author contributions

**Data curation:** Zhen Li.

**Funding acquisition:** Dan Tang, Jiguang Guo.

**Investigation:** Dan Tang.

**Project administration:** JianBin Zhang.

**Writing – original draft:** JianBin Zhang.

**Writing – review and editing:** JianBin Zhang, Jiguang Guo.
